# 1-{(*E*)-[(2-Fluoro-5-nitro­phen­yl)imino]­meth­yl}naphthalen-2-ol

**DOI:** 10.1107/S1600536813012099

**Published:** 2013-05-11

**Authors:** Alan R. Kennedy, Mehmet Akkurt, Antar A. Abdelhamid, Shaaban K. Mohamed, Gary J. Miller

**Affiliations:** a Department of Pure & Applied Chemistry, University of Strathclyde, 295 Cathedral Street, Glasgow G1 1XL, Scotland; b Department of Physics, Faculty of Sciences, Erciyes University, 38039 Kayseri, Turkey; c Chemistry and Environmental Division, Manchester Metropolitan University, Manchester, M1 5GD, England; d Chemistry Department, Faculty of Sccience, Mini University, 61519 El-Minia, Egypt; e Analytical Sciences, Manchester Metropolitan University, Manchester, M1 5GD, England

## Abstract

The title mol­ecule, C_17_H_11_FN_2_O_3_, is nearly planar [maximum deviation = 0.197 (1) Å] and the mol­ecular conformation is stabilized by an N—H⋯O hydrogen bond forming an *S*(6) ring motif. The H atom of the intra­molecular hydrogen bond was found to be disordered over two sites and thus both the hy­droxy and keto tautomers are simultaneously present in the solid. Refinement of the occupancy of this site suggests that the hy­droxy form is the major component [occupancy refined to 0.59 (3):0.41 (3)]. Bond lengths are also largely consistent with dominance of the hy­droxy form. In the crystal, mol­ecules are linked by C—H⋯O hydrogen bonds, forming layers parallel to (101). π–π stacking inter­actions [centroid–centroid distances = 3.5649 (9) and 3.7579 (9) Å] inter-connect these layers.

## Related literature

For the broad range of biological applications of Schiff bases, see, for example: Cozzi (2004 ▶); Chandra & Sangeetika (2004 ▶); Sari *et al.* (2003 ▶); Verma *et al.* (2004 ▶). For the significance of fluorine atoms in drug structures, see: Blair *et al.* (2000 ▶); Kirk *et al.* (1979 ▶); LeBars *et al.* (1987 ▶). For a related structure, see: Akkurt *et al.* (2012 ▶). For hydrogen-bond motifs, see: Bernstein *et al.* (1995 ▶). 
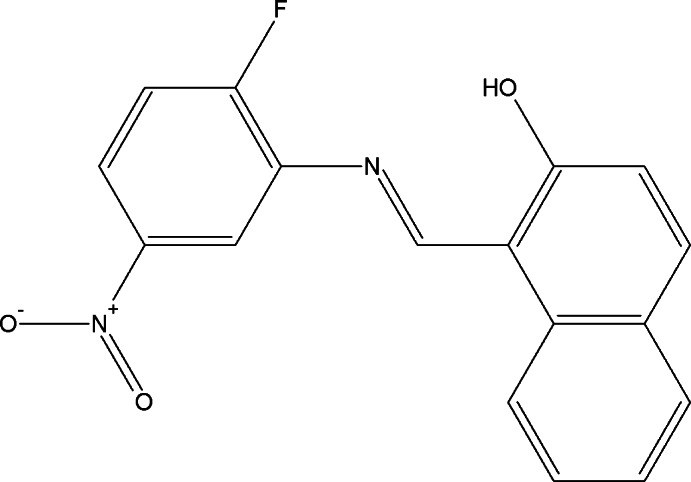


## Experimental

### 

#### Crystal data

C_17_H_11_FN_2_O_3_
*M* *_r_* = 310.28
Monoclinic, 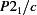

*a* = 14.2226 (6) Å
*b* = 13.0856 (5) Å
*c* = 7.3801 (3) Å
β = 94.151 (4)°
*V* = 1369.92 (10) Å^3^
*Z* = 4
Mo *K*α radiation
μ = 0.11 mm^−1^
*T* = 123 K
0.5 × 0.2 × 0.05 mm

#### Data collection

Oxford Diffraction Xcalibur Eos diffractometer
Absorption correction: multi-scan (*CrysAlis PRO*; Oxford Diffraction, 2010 ▶) *T*_min_ = 0.973, *T*_max_ = 0.994
15943 measured reflections
4043 independent reflections
3166 reflections with *I* > 2σ(*I*)
*R*_int_ = 0.036

#### Refinement

*R*[*F* ^2^ > 2σ(*F* ^2^)] = 0.052
*wR*(*F* ^2^) = 0.135
*S* = 1.07
4043 reflections
215 parameters
2 restraints
H atoms treated by a mixture of independent and constrained refinement
Δρ_max_ = 0.33 e Å^−3^
Δρ_min_ = −0.27 e Å^−3^

### 

Data collection: *CrysAlis PRO* (Oxford Diffraction, 2010 ▶); cell refinement: *CrysAlis PRO*; data reduction: *CrysAlis PRO*; program(s) used to solve structure: *SHELXS97* (Sheldrick, 2008 ▶); program(s) used to refine structure: *SHELXL97* (Sheldrick, 2008 ▶); molecular graphics: *ORTEP-3 for Windows* (Farrugia, 2012 ▶); software used to prepare material for publication: *WinGX* (Farrugia, 2012 ▶) and *PLATON* (Spek, 2009 ▶).

## Supplementary Material

Crystal structure: contains datablock(s) global, I. DOI: 10.1107/S1600536813012099/sj5319sup1.cif

Structure factors: contains datablock(s) I. DOI: 10.1107/S1600536813012099/sj5319Isup2.hkl

Supplementary material file. DOI: 10.1107/S1600536813012099/sj5319Isup3.cml

Additional supplementary materials:  crystallographic information; 3D view; checkCIF report

## Figures and Tables

**Table 1:** Hydrogen-bond geometry (Å, °)

*D*—H⋯*A*	*D*—H	H⋯*A*	*D*⋯*A*	*D*—H⋯*A*
O1—H1⋯N1	0.88 (1)	1.68 (2)	2.4969 (16)	152 (4)
C3—H3⋯O3^i^	0.95	2.48	3.222 (2)	135
C14—H14⋯O1^ii^	0.95	2.35	3.1724 (19)	145

Symmetry codes: (i) 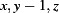; (ii) 
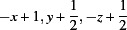
.

**Fig. 1.**
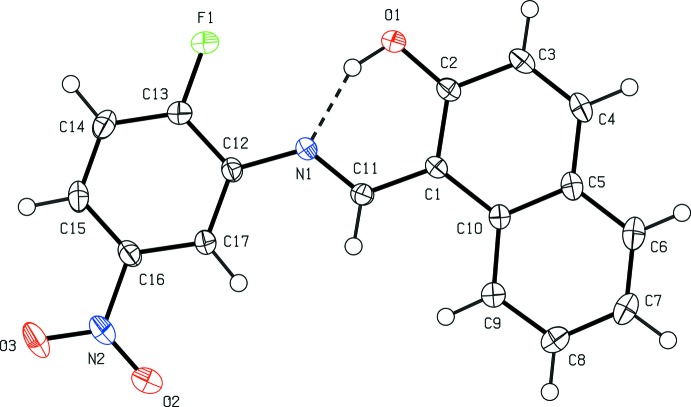
The title compound with the atom numbering scheme. Displacement ellipsoids for non-H atoms are drawn at the 50% probability level. Only the major component (OH tautomer) of the disordered OH and NH tautomers is shown. The intramolecular hydrogen bond is drawn as a dashed line.

**Fig. 2.**
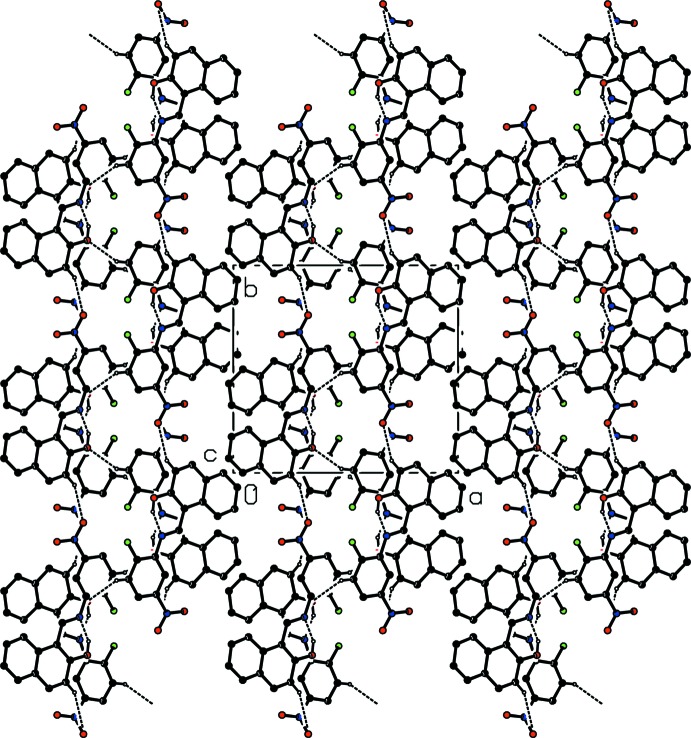
The hydrogen bonding of the title compound viewed along the c axis. The minor component (NH tautomer) of disorder and H atoms not involved in hydrogen bonding are omitted for clarity. Hydrogen bonds are drawn as dashed lines.

**Fig. 3.**
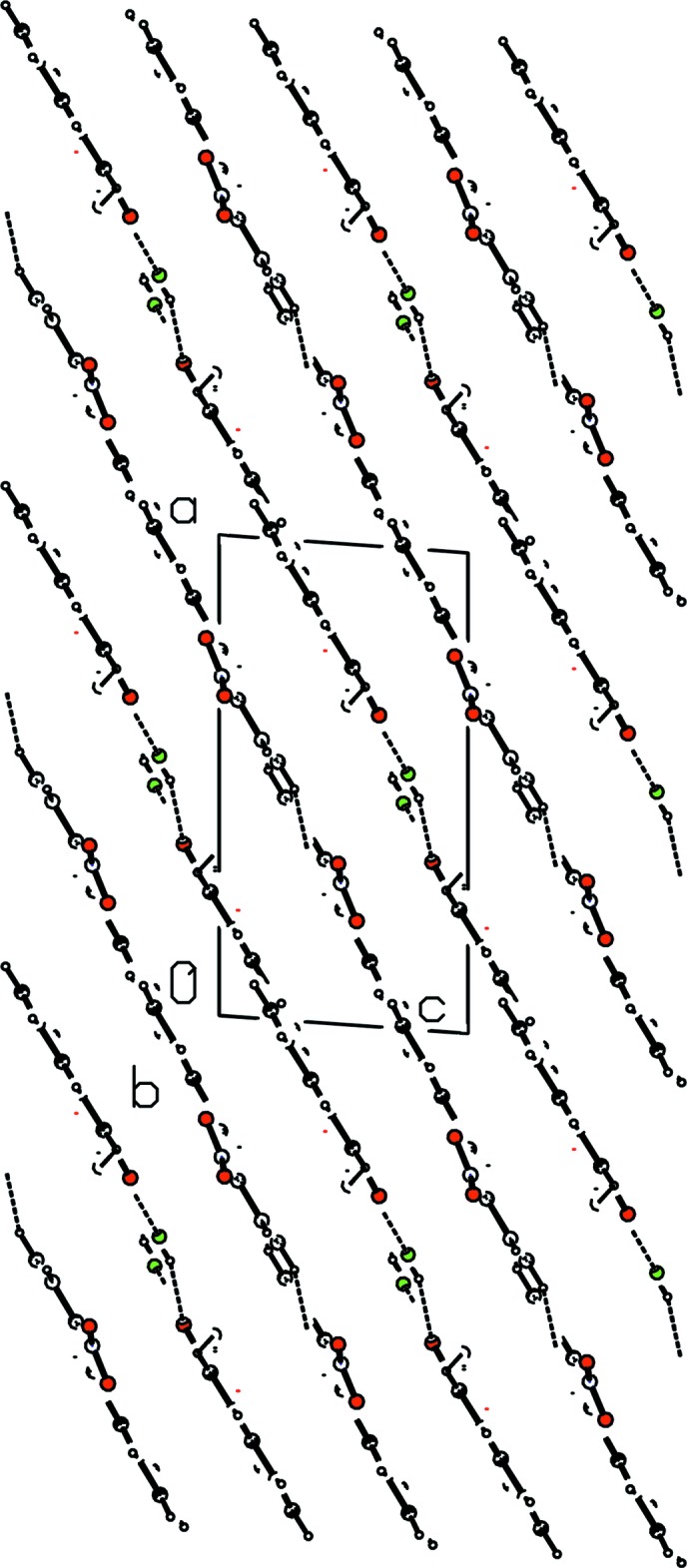
The molecular packing of the title compound viewed along the b axis, showing the two dimensional layers parallel to (101). Hydrogen bonds are drawn as dashed lines.

## supplementary crystallographic information

### Comment

Schiff bases have been widely studied due to their importance in industrial and biological applications. They serve for example as, antibacterial, antifungal, anticancer (Sari *et al.*, 2003, Verma *et al.*, 2004) and herbicidal agents (Cozzi, 2004; Chandra & Sangeetika, 2004). It is well known that the introduction of fluorine atom into an organic molecule causes dramatic changes in its biological profile (Blair *et al.*, 2000), mainly due to the high electronegativity of fluorine. Incorporating fluorine increases fat solubility, improving the drug's partitioning into membranes and hence increasing bioavailability (LeBars *et al.*, 1987). Fluorination can also aid hydrophobic interactions between the drug and binding sites on receptors or enzymes (Kirk *et al.*, 1979). Further to our study in synthesis of fluorinated bioactive compounds we herein report the synthesis and crystal structure of the title compound.

As seen in Fig. 1, the title molecule (I) is nearly planar with maximum deviations of 0.197 (1) Å for O3, -0.157 (1) Å for C9 and 0.145 (2) Å for C6. The napthalene ring system (C1–C10) makes a dihedral angle of 5.04 (6) ° with the the benzene ring (C12–C17) of the 1-fluoro-4-nitrobenzene group. The C1–C11–N1–C12, F1–C13–C12–N1, O1–C2–C1–C11, O2–N2–C16—C17 and O3–N2–C16 C15 torsion angles are -179.56 (13), -179.39 (13), 2.4 (2), -8.9 (2) and -9.7 (2) °, respectively. All bond lengths and angles are similar to those of a related structure previously reported (Akkurt *et al.*, 2012).

An N—H···O hydrogen bond stabilizes the molecular conformation of (I) forming an S(6) ring motif (Bernstein *et al.*, 1995; Fig. 1). In the crystal structure, C—H···O hydrogen bonds (Table 1, Fig. 2) link the molecules to each other, forming two dimensional layers parallel to (101) (Fig. 3). In addition, these layers connect to each other by π-π stacking interactions [*Cg*1···*Cg*3*^iii^* = 3.5649 (9) Å and *Cg*3···*Cg*3*^iv^* = 3.7579 (9) Å; where symmetry codes (*iii*) = *x*, 1/2 - *y*, 1/2 + *z* and (*iv*) = 1 - *x*, 1 - *y*, 1 - *z*; *Cg*1 and *Cg*3 are the centroids of the C1–C5/C10 and C12–C17 benzene rings, respectively].

### Experimental

The title compound was obtained unintentionally in a good yield from a three components reaction by heating of 1 mmol (172 mg) 2-hydroxynaphthalene-1-carbaldehyde, 1 mmol (156 mg) 2-fluoro-5-nitroaniline and 1 mmol (188 mg) 5-phenylcyclohexane-1,3-dione in ethanol for 8 h at 350 K. The solvent was evaporated under *vacuum* and the resulting solid was crystallized from a mixture of ethanol and few drops of acetone. Yellow rods of product (*M*.p. 471 K) were collected (73% yield) of sufficient quality for X-ray diffraction.

### Refinement

Difference synthesis suggested a disordered model for H1 was appropriate. Refinement over two sites with thermal displacement ellipsoids constrained to be equal and with both O1—H1 and N1—H2 distances restrained to 0.88 (1) A gave a model with 0.59 (3):0.41 (3) site occupancy in favour of the OH form. The C-bound H atoms were placed in geometrically optimized positions and constrained to ride on their parent atoms with C—H = 0.95 (aromatic) Å and with *U*_iso_(H) = 1.2*U*_eq_(C).

### Crystal data

**Table:**

C_17_H_11_FN_2_O_3_	*F*(000) = 640
*M**_r_* = 310.28	*D*_x_ = 1.504 Mg m^−^^3^
Monoclinic, *P*2_1_/*c*	Mo *K*α radiation, λ = 0.71073 Å
Hall symbol: -P 2ybc	Cell parameters from 5692 reflections
*a* = 14.2226 (6) Å	θ = 3.2–30.7°
*b* = 13.0856 (5) Å	µ = 0.11 mm^−^^1^
*c* = 7.3801 (3) Å	*T* = 123 K
β = 94.151 (4)°	Cut rod, yellow
*V* = 1369.92 (10) Å^3^	0.5 × 0.2 × 0.05 mm
*Z* = 4	

### Data collection

**Table:**

Oxford Diffraction Xcalibur Eos diffractometer	4043 independent reflections
Radiation source: Enhance (Mo) X-ray Source	3166 reflections with *I* > 2σ(*I*)
Graphite monochromator	*R*_int_ = 0.036
Detector resolution: 16.0727 pixels mm^-1^	θ_max_ = 30.8°, θ_min_ = 3.2°
ω scans	*h* = −19→20
Absorption correction: multi-scan (*CrysAlis PRO*; Oxford Diffraction, 2010)	*k* = −18→18
*T*_min_ = 0.973, *T*_max_ = 0.994	*l* = −10→10
15943 measured reflections	

### Refinement

**Table:**

Refinement on *F*^2^	Primary atom site location: structure-invariant direct methods
Least-squares matrix: full	Secondary atom site location: difference Fourier map
*R*[*F*^2^ > 2σ(*F*^2^)] = 0.052	Hydrogen site location: inferred from neighbouring sites
*wR*(*F*^2^) = 0.135	H atoms treated by a mixture of independent and constrained refinement
*S* = 1.07	*w* = 1/[σ^2^(*F*_o_^2^) + (0.0528*P*)^2^ + 0.7073*P*] where *P* = (*F*_o_^2^ + 2*F*_c_^2^)/3
4043 reflections	(Δ/σ)_max_ < 0.001
215 parameters	Δρ_max_ = 0.33 e Å^−^^3^
2 restraints	Δρ_min_ = −0.27 e Å^−^^3^

### Special details

**Table:**

Geometry. Bond distances, angles *etc*. have been calculated using the rounded fractional coordinates. All su's are estimated from the variances of the (full) variance-covariance matrix. The cell e.s.d.'s are taken into account in the estimation of distances, angles and torsion angles
Refinement. Refinement on *F*^2^ for ALL reflections except those flagged by the user for potential systematic errors. Weighted *R*-factors *wR* and all goodnesses of fit *S* are based on *F*^2^, conventional *R*-factors *R* are based on *F*, with *F* set to zero for negative *F*^2^. The observed criterion of *F*^2^ > σ(*F*^2^) is used only for calculating -*R*-factor-obs *etc*. and is not relevant to the choice of reflections for refinement. *R*-factors based on *F*^2^ are statistically about twice as large as those based on *F*, and *R*-factors based on ALL data will be even larger.

### Fractional atomic coordinates and isotropic or equivalent isotropic displacement parameters (Å^2^)

**Table:**

	*x*	*y*	*z*	*U*_iso_*/*U*_eq_	Occ. (<1)
F1	0.46913 (7)	0.33478 (7)	0.24068 (14)	0.0300 (3)	
O1	0.35134 (8)	0.12068 (8)	0.35743 (15)	0.0236 (3)	
O2	0.21721 (11)	0.66754 (10)	0.5527 (3)	0.0535 (6)	
O3	0.33368 (11)	0.75984 (10)	0.4783 (2)	0.0488 (5)	
N1	0.31672 (9)	0.30387 (9)	0.41994 (17)	0.0185 (3)	
N2	0.29390 (11)	0.67768 (10)	0.4896 (2)	0.0313 (4)	
C1	0.21630 (10)	0.17180 (10)	0.51171 (19)	0.0171 (3)	
C2	0.27574 (10)	0.09654 (11)	0.44206 (19)	0.0191 (4)	
C3	0.25569 (11)	−0.00890 (11)	0.4642 (2)	0.0228 (4)	
C4	0.18177 (11)	−0.03893 (11)	0.5579 (2)	0.0240 (4)	
C5	0.11990 (11)	0.03344 (11)	0.6319 (2)	0.0215 (4)	
C6	0.04339 (12)	0.00082 (13)	0.7289 (2)	0.0280 (5)	
C7	−0.01858 (11)	0.06974 (13)	0.7933 (2)	0.0279 (5)	
C8	−0.00642 (11)	0.17414 (13)	0.7622 (2)	0.0258 (4)	
C9	0.06885 (10)	0.20868 (12)	0.6710 (2)	0.0221 (4)	
C10	0.13476 (10)	0.13968 (11)	0.60456 (19)	0.0176 (3)	
C11	0.24075 (10)	0.27705 (11)	0.49616 (18)	0.0177 (3)	
C12	0.34637 (10)	0.40451 (10)	0.39717 (19)	0.0181 (3)	
C13	0.42636 (10)	0.41877 (11)	0.3009 (2)	0.0212 (4)	
C14	0.46365 (11)	0.51311 (13)	0.2649 (2)	0.0251 (4)	
C15	0.42032 (11)	0.59926 (12)	0.3290 (2)	0.0247 (4)	
C16	0.34099 (11)	0.58627 (11)	0.4245 (2)	0.0226 (4)	
C17	0.30302 (10)	0.49186 (11)	0.4606 (2)	0.0200 (4)	
H1	0.357 (3)	0.1877 (8)	0.365 (5)	0.0350*	0.59 (3)
H3	0.29440	−0.05880	0.41300	0.0270*	
H4	0.17090	−0.10980	0.57460	0.0290*	
H6	0.03470	−0.07010	0.74980	0.0340*	
H7	−0.06960	0.04670	0.85880	0.0330*	
H8	−0.05030	0.22180	0.80420	0.0310*	
H9	0.07650	0.28000	0.65260	0.0260*	
H11	0.20120	0.32800	0.54190	0.0210*	
H14	0.51790	0.51890	0.19770	0.0300*	
H15	0.44440	0.66560	0.30790	0.0300*	
H17	0.24850	0.48650	0.52710	0.0240*	
H2	0.350 (3)	0.253 (3)	0.380 (6)	0.0280*	0.41 (3)

### Atomic displacement parameters (Å^2^)

**Table:**

	*U*^11^	*U*^22^	*U*^33^	*U*^12^	*U*^13^	*U*^23^
F1	0.0281 (5)	0.0236 (5)	0.0402 (6)	0.0041 (4)	0.0155 (4)	−0.0009 (4)
O1	0.0250 (5)	0.0173 (5)	0.0298 (6)	0.0025 (4)	0.0100 (4)	−0.0002 (4)
O2	0.0504 (9)	0.0238 (7)	0.0908 (12)	0.0049 (6)	0.0365 (9)	−0.0039 (7)
O3	0.0531 (9)	0.0143 (6)	0.0801 (11)	−0.0041 (6)	0.0128 (8)	−0.0018 (6)
N1	0.0198 (6)	0.0141 (6)	0.0220 (6)	0.0010 (4)	0.0038 (5)	0.0016 (4)
N2	0.0368 (8)	0.0152 (6)	0.0423 (8)	0.0018 (6)	0.0056 (7)	0.0009 (6)
C1	0.0186 (6)	0.0139 (6)	0.0187 (6)	0.0012 (5)	0.0011 (5)	−0.0005 (5)
C2	0.0217 (7)	0.0161 (6)	0.0193 (6)	0.0021 (5)	0.0007 (5)	0.0000 (5)
C3	0.0282 (8)	0.0148 (7)	0.0253 (7)	0.0024 (5)	0.0012 (6)	−0.0027 (5)
C4	0.0292 (8)	0.0141 (6)	0.0282 (8)	−0.0028 (6)	−0.0015 (6)	0.0004 (6)
C5	0.0227 (7)	0.0177 (7)	0.0236 (7)	−0.0033 (5)	−0.0012 (6)	0.0026 (5)
C6	0.0282 (8)	0.0256 (8)	0.0303 (8)	−0.0075 (6)	0.0023 (6)	0.0062 (6)
C7	0.0231 (7)	0.0336 (9)	0.0274 (8)	−0.0065 (6)	0.0051 (6)	0.0055 (7)
C8	0.0209 (7)	0.0309 (8)	0.0259 (7)	−0.0003 (6)	0.0048 (6)	−0.0006 (6)
C9	0.0220 (7)	0.0199 (7)	0.0246 (7)	−0.0004 (6)	0.0040 (6)	0.0003 (6)
C10	0.0186 (6)	0.0166 (6)	0.0175 (6)	−0.0021 (5)	0.0005 (5)	0.0009 (5)
C11	0.0179 (6)	0.0165 (6)	0.0186 (6)	0.0012 (5)	0.0016 (5)	−0.0009 (5)
C12	0.0179 (6)	0.0150 (6)	0.0213 (6)	−0.0006 (5)	0.0002 (5)	0.0023 (5)
C13	0.0204 (7)	0.0200 (7)	0.0234 (7)	0.0015 (5)	0.0040 (6)	−0.0001 (5)
C14	0.0196 (7)	0.0282 (8)	0.0280 (8)	−0.0043 (6)	0.0047 (6)	0.0049 (6)
C15	0.0240 (7)	0.0194 (7)	0.0305 (8)	−0.0062 (6)	0.0002 (6)	0.0061 (6)
C16	0.0249 (7)	0.0149 (7)	0.0279 (7)	0.0005 (5)	0.0006 (6)	0.0007 (5)
C17	0.0205 (7)	0.0159 (6)	0.0239 (7)	−0.0008 (5)	0.0036 (5)	0.0020 (5)

### Geometric parameters (Å, º)

**Table:**

F1—C13	1.3471 (17)	C8—C9	1.381 (2)
O1—C2	1.3203 (18)	C9—C10	1.415 (2)
O2—N2	1.224 (2)	C12—C17	1.396 (2)
O3—N2	1.2206 (19)	C12—C13	1.397 (2)
O1—H1	0.882 (12)	C13—C14	1.377 (2)
N1—C12	1.3967 (18)	C14—C15	1.384 (2)
N1—C11	1.3019 (19)	C15—C16	1.383 (2)
N2—C16	1.469 (2)	C16—C17	1.382 (2)
N1—H2	0.88 (4)	C3—H3	0.9500
C1—C11	1.427 (2)	C4—H4	0.9500
C1—C2	1.418 (2)	C6—H6	0.9500
C1—C10	1.451 (2)	C7—H7	0.9500
C2—C3	1.421 (2)	C8—H8	0.9500
C3—C4	1.358 (2)	C9—H9	0.9500
C4—C5	1.428 (2)	C11—H11	0.9500
C5—C10	1.423 (2)	C14—H14	0.9500
C5—C6	1.411 (2)	C15—H15	0.9500
C6—C7	1.370 (2)	C17—H17	0.9500
C7—C8	1.398 (2)		
			
C2—O1—H1	106 (3)	F1—C13—C12	117.56 (12)
C11—N1—C12	124.99 (13)	F1—C13—C14	118.58 (13)
O2—N2—C16	118.44 (13)	C12—C13—C14	123.86 (13)
O3—N2—C16	118.06 (15)	C13—C14—C15	118.48 (14)
O2—N2—O3	123.49 (15)	C14—C15—C16	118.27 (14)
C11—N1—H2	115 (3)	N2—C16—C15	118.34 (13)
C12—N1—H2	120 (3)	N2—C16—C17	118.12 (14)
C10—C1—C11	121.68 (12)	C15—C16—C17	123.54 (14)
C2—C1—C11	119.07 (13)	C12—C17—C16	118.64 (13)
C2—C1—C10	119.19 (12)	C2—C3—H3	120.00
C1—C2—C3	120.18 (13)	C4—C3—H3	120.00
O1—C2—C3	117.64 (13)	C3—C4—H4	119.00
O1—C2—C1	122.17 (13)	C5—C4—H4	119.00
C2—C3—C4	120.59 (14)	C5—C6—H6	119.00
C3—C4—C5	121.62 (13)	C7—C6—H6	119.00
C4—C5—C6	120.84 (14)	C6—C7—H7	120.00
C4—C5—C10	119.46 (13)	C8—C7—H7	120.00
C6—C5—C10	119.70 (14)	C7—C8—H8	120.00
C5—C6—C7	121.06 (15)	C9—C8—H8	120.00
C6—C7—C8	119.74 (15)	C8—C9—H9	119.00
C7—C8—C9	120.61 (15)	C10—C9—H9	119.00
C8—C9—C10	121.10 (14)	N1—C11—H11	120.00
C1—C10—C5	118.81 (13)	C1—C11—H11	120.00
C5—C10—C9	117.75 (13)	C13—C14—H14	121.00
C1—C10—C9	123.44 (13)	C15—C14—H14	121.00
N1—C11—C1	120.63 (13)	C14—C15—H15	121.00
N1—C12—C17	125.97 (13)	C16—C15—H15	121.00
N1—C12—C13	116.83 (12)	C12—C17—H17	121.00
C13—C12—C17	117.20 (13)	C16—C17—H17	121.00
			
C11—N1—C12—C17	3.8 (2)	C10—C5—C6—C7	1.8 (2)
C12—N1—C11—C1	179.56 (13)	C6—C5—C10—C1	177.21 (13)
C11—N1—C12—C13	−175.67 (14)	C4—C5—C6—C7	−177.11 (14)
O3—N2—C16—C15	−9.7 (2)	C4—C5—C10—C9	176.47 (14)
O3—N2—C16—C17	170.89 (15)	C6—C5—C10—C9	−2.4 (2)
O2—N2—C16—C15	170.52 (17)	C5—C6—C7—C8	0.3 (2)
O2—N2—C16—C17	−8.9 (2)	C6—C7—C8—C9	−1.6 (2)
C10—C1—C2—C3	−0.4 (2)	C7—C8—C9—C10	0.9 (2)
C2—C1—C11—N1	−0.3 (2)	C8—C9—C10—C1	−178.49 (14)
C10—C1—C2—O1	−179.51 (13)	C8—C9—C10—C5	1.1 (2)
C11—C1—C10—C9	6.1 (2)	N1—C12—C13—C14	179.10 (14)
C2—C1—C10—C5	3.5 (2)	C17—C12—C13—F1	179.39 (13)
C11—C1—C2—O1	−2.4 (2)	C17—C12—C13—C14	−0.5 (2)
C11—C1—C2—C3	176.71 (13)	N1—C12—C13—F1	−1.1 (2)
C11—C1—C10—C5	−173.49 (13)	N1—C12—C17—C16	−179.28 (14)
C10—C1—C11—N1	176.69 (13)	C13—C12—C17—C16	0.2 (2)
C2—C1—C10—C9	−176.87 (14)	F1—C13—C14—C15	−179.15 (13)
C1—C2—C3—C4	−2.5 (2)	C12—C13—C14—C15	0.7 (2)
O1—C2—C3—C4	176.68 (14)	C13—C14—C15—C16	−0.7 (2)
C2—C3—C4—C5	2.2 (2)	C14—C15—C16—N2	−178.86 (14)
C3—C4—C5—C10	1.1 (2)	C14—C15—C16—C17	0.5 (2)
C3—C4—C5—C6	179.98 (15)	N2—C16—C17—C12	179.10 (13)
C4—C5—C10—C1	−3.9 (2)	C15—C16—C17—C12	−0.3 (2)

### Hydrogen-bond geometry (Å, º)

**Table:**

*D*—H···*A*	*D*—H	H···*A*	*D*···*A*	*D*—H···*A*
O1—H1···N1	0.88 (1)	1.68 (2)	2.4969 (16)	152 (4)
C3—H3···O3^i^	0.95	2.48	3.222 (2)	135
C14—H14···O1^ii^	0.95	2.35	3.1724 (19)	145

Symmetry codes: (i) *x*, *y*−1, *z*; (ii) −*x*+1, *y*+1/2, −*z*+1/2.
